# Case of Puerperal Convulsions

**Published:** 1851-09

**Authors:** J. A. Preston

**Affiliations:** Hartland, Wisconsin


*Case of Puerperal Convulsions.* By J. A. PRESTON, M. D., of Hartland, Wisconsin.

Mrs. ——— was taken in labor Thursday morning, July 24. I was sent for and arrived about 3 o'clock P. M. As the pains were then slight, no examination was made until 5 o'clock. At this time the pains become more frequent and severe, promising a speedy accomplishment of the labor. The os uteri was well dilated, the head presenting in the first position and occupying the superior strait.

The patient, whose age was 28, was primaparous, possessed a well marked nervous temperament, with a strong predisposition to cerebral determination. During the night, the pains continued at quite regular intervals, but were wanting in strength and efficiency. Labor went on well, or at least without any unusual complication, until 2 o'clock Friday morning, when she was seized with a very severe convulsion. So far as I was able to ascertain, there was not the slightest premonition of the phenomena which followed. After using such immediate measures as seemed to me proper, I sent for such counsel as I knew to be provided with forceps. I have a pair of Bedford's beautiful instruments, but the distance to my house determined me to send for the nearest assistance. A second convulsion more severe than the first induced me to decline the hazard of waiting for means which might come too late; I therefore took the responsibility of delivering the child forcibly.

Up to this time my efforts had been directed entirely toward equalizing the circulation. Sinipisms were applied to the feet, ankles and arms, and cold water was poured from a height upon the head. Bleeding did not appear to be indicated, and as it was subsequently practiced without good results, I think no benefit would have accrued from its earlier adoption. Yielding to the conviction that nothing but immediate delivery would avert a fatal issue, I perforated the fœtal cranium, and delivered without difficulty a dead male child. The result did not justify my anticipations, as the convulsions continued apparently, though probably not really unaffected by the delivery.

Of this part of my practice, I shall attempt no further defence, than the foregoing statement of facts. I can only say that under the circumstances I yielded to what I conscientiously believed was an imperative necessity. After the arrival of counsel the same remedial measures were continued vigorously, and after a third or fourth convulsion as the pulse became more fully developed, the patient was bled ℥x. It was deemed advisable also to give Assafœtida, but from the excessive irritability of the stomach it was rejected and not again repeated. At this time it was proposed to try the effect of Opium internally, but the proposition was overruled by counsel. The convulsions continued for twelve hours with little abatement, the patient lying in a comatose state between the paroxysms. The water treatment was continued, and the bowels had been moved by emmata of Castor Oil, Turpentine and Milk. At this time, as the patient's strength seemed rapidly waning, the case was looked upon as hopeless; this opinion was concurr ed in by other intelligent counsel which had arrived. It was determined, however, as a *derrnier* resort, to try the effect of the warm wet blanket, and a liberal dose of Opium per anum. The warm blanket was applied, grs. v. of Haskell and Merrick's Powdered Opium administered by injection, and developments anxiously awaited. Soon after the exhibition of the opium, which was some two hours after the occurrence of a paroxysm, there was a pretty severe convulsion, though from the brief interval which had elapsed the primary operation of the narcotic could not have been the exciting cause.

There was only one or two more slight convulsions, making some twelve in all. The pulse grew firmer and steadier as the action of the opiate became manifest, the skin was bedewed with copious moisture and refreshing sleep followed. The lochial discharge, aided by the water treatment, was duly established, lactation commenced on the fourth day, and during the week which included the whole period of my attendance, there was not the slightest evidence of fever or visceral inflammation. This happy result, I have no doubt, was chiefly attributable to the sustaining power of the Opium, and the equalizing influence of the general fomentation.

The result of the case as regards the mother, certainly, was most happy, and under all the circumstances—though I would scorn to screen myself from deserved censure—I can hardly feel called upon to regret any part of my course. It has caused severe animadversion by consequence of misrepresentations, made no doubt to answer ulterior purposes. But the cause of truth will never suffer *permanently*, by the aspersions of malice. Candor may sometimes compel us to make confessions little flattering to personal vanity, but self aggrandizement is not the legitimate aim of philosophy. The physician who cannot afford to confess a wrong must have but few successes to boast. Hippocrates has had the honesty to confess that he once mistook a suture for a fracture of the skull—a confession which Cullen remarks does him more credit than his most brilliant discovery.

*August* 13, 1851.

[The good effects of Chloroform and Ether in cases of the description given above, and the promptness of their action, have commended their use to the favorable consideration of the profession; and where they have been tried it has generally been with the happiest effects. In a large proportion of the cases, their use and the Anodyne treatment are plainly indicated.—Ed.]

